# Transformational leadership, empowerment, and job satisfaction: the mediating role of employee empowerment

**DOI:** 10.1186/s12960-016-0171-2

**Published:** 2016-12-01

**Authors:** Sang Long Choi, Chin Fei Goh, Muhammad Badrull Hisyam Adam, Owee Kowang Tan

**Affiliations:** 1 Raffles University Iskandar, Menara Kotaraya, Jalan Trus, 80000 Johor Bahru, Johor Malaysia; 2 Faculty of Management, Universiti Teknologi Malaysia, 81310 Johor Bahru, Johor Malaysia; 3 International Business School, Universiti Teknologi Malaysia, 54100 Kuala Lumpur, Malaysia

**Keywords:** Transformational leadership, Job satisfaction, Empowerment, Nurse, Healthcare management

## Abstract

**Background:**

Recent studies have revealed that nursing staff turnover remains a major problem in emerging economies. In particular, nursing staff turnover in Malaysia remains high due to a lack of job satisfaction. Despite a shortage of healthcare staff, the Malaysian government plans to create 181 000 new healthcare jobs by 2020 through the Economic Transformation Programme (ETP). This study investigated the causal relationships among perceived transformational leadership, empowerment, and job satisfaction among nurses and medical assistants in two selected large private and public hospitals in Malaysia. This study also explored the mediating effect of empowerment between transformational leadership and job satisfaction.

**Methods:**

This study used a survey to collect data from 200 nursing staff, i.e., nurses and medical assistants, employed by a large private hospital and a public hospital in Malaysia. Respondents were asked to answer 5-point Likert scale questions regarding transformational leadership, employee empowerment, and job satisfaction. Partial least squares-structural equation modeling (PLS-SEM) was used to analyze the measurement models and to estimate parameters in a path model. Statistical analysis was performed to examine whether empowerment mediated the relationship between transformational leadership and job satisfaction.

**Results:**

This analysis showed that empowerment mediated the effect of transformational leadership on the job satisfaction in nursing staff. Employee empowerment not only is indispensable for enhancing job satisfaction but also mediates the relationship between transformational leadership and job satisfaction among nursing staff.

**Conclusions:**

The results of this research contribute to the literature on job satisfaction in healthcare industries by enhancing the understanding of the influences of empowerment and transformational leadership on job satisfaction among nursing staff. This study offers important policy insight for healthcare managers who seek to increase job satisfaction among their nursing staff.

## Background

In September 2010, the Economic Transformation Programme (ETP) was launched by the Malaysian government as part of the National Transformation Programme to achieve a self-sufficient, developed nation by 2020 [1]. The ETP was established to develop 12 National Key Economic Areas (NKEAs) to elevate gross national income per capita to US$15 000. Healthcare is one of the 12 NKEAs covered by the ETP. Changing demographics, an aging population, increasingly health-conscious lifestyles, and an affluent society have strengthened the rapidly growing healthcare sector in Malaysia [1]. The Malaysian government plans to create 181 000 new healthcare jobs by 2020 through the ETP [2].

In general, the Ministry of Health Malaysia has overall responsibilities to formulate policies for human resources regarding health [3]. The Ministry of Health acknowledges that it is of upmost importance to address human capital development in the Malaysian healthcare sector [3, 4]. An adequate and competent workforce is vital to improving the delivery of healthcare services by reducing the probability of medical errors and improving service quality, among other benefits. In this regard, the shortage of human resources is the main problem experienced by the healthcare sector in Malaysia [5, 6]. Specifically, the shortage of healthcare professionals who can provide skilled nursing services is a major challenge. In this study, we regard registered nurses and medical assistants as nursing staff [5]. In Malaysia, medical assistants are similar to nurse practitioners in other countries [7]. In addition to nursing services, medical assistants are also qualified to handle patients with simple acute conditions [8]. Nurses and medical assistants are required by law to undertake a different education program. Additionally, medical assistants and nurses are required to register under the respective statutory board before being eligible to practice. Owing to historical and cultural reasons, only females were eligible to become registered nurses, whereas males were only permitted to register as medical assistants until early 2000 [3, 5]. The first batch of male nurses and female medical assistants were registered under their corresponding boards in the years 2008 and 2009, respectively [3]. However, nurses and medical assistants who intend to specialize in various specialties are assessed through credentialing in four areas: (1) intensive care, (2) perioperative care, (3) ophthalmology, and (4) emergency medicine and trauma care.

Similar to other Islamic nations, nursing staff in Malaysia are dominated by women and have a lower status compared to their Western counterparts [9]. The current nursing and health system in Malaysia is still largely the same as with the old system that was introduced during the British colonial era [9]. A study in Malaysia has shown that the majority of nurses can be described as ignorant of their oppressed status, exhibiting “unquestioning acceptance of the role of nurses, the power of the system, and the dominance of physicians” [9]. Multiple traditional and cultural factors have adversely influenced the status of nursing in Malaysia [9, 10]. Among the primary reasons for the indigent status of nurses are the lack of public recognition, low educational entry requirements, and unfavorable employment conditions, including low salaries and poor working conditions. Such oppression adversely affects the job satisfaction of nursing staff in Malaysia [4, 9, 11]. Furthermore, nurses are often victimized as the root cause of declining nursing services in Malaysia while some important organizational factors, such as nursing shortage, lack of support, and poor working conditions, are overlooked [5]. The nursing shortage is evident from the 2010 report from the Malaysian Ministry of Health, which stated that the density of local nurses is 1.35 per 1000 people, which is 47.3% lower than the global density of nurses. Several reports have consistently noted that the high turnover of nurses is due to low job satisfaction in Malaysia [4, 12, 13]. Although no study to date has measured job satisfaction among medical assistants in Malaysia, we believe that they have low job satisfaction because of their job responsibilities in the nursing services. Therefore, job satisfaction among nursing staff is a main challenge for the Malaysian government in order to reform the healthcare sector through the ETP.

Traditional hierarchical structures in hospitals have resulted in medical dominance and suppressed nursing and other health professional in clinical environments [9]. Such problems are a major challenge in Islamic nations, including Malaysia. Recently, several scholars have suggested that a new form of healthcare leadership and empowerment is required to improve the job satisfaction of nursing staff in Malaysia [9, 11, 14]. The transactional style of leadership that pervades the healthcare institutions is believed to be a root cause of the nursing staff turnover rate [14]. Thus, transformational leadership can be adopted to complement the existing transactional leadership in healthcare institutions. Our review also shows that nursing management studies have offered preliminary evidence to support the aforementioned suggestions in the Malaysian context. First, several studies have found that transformational leadership is positively correlated with job satisfaction among nurses in Malaysia [15, 16]. Second, prior studies have shown that empowerment is positively related to job satisfaction among nurses in Malaysia and in several other countries [11, 17, 18]. Furthermore, qualitative analysis has shown that empowerment could be a promising solution to restructuring the work environment and to reduce the powerlessness senses among nursing staff in Malaysia [9, 11, 12]. Nevertheless, this empirical evidence is fragmented, owing to a lack of comprehensive studies that investigate transformational leadership, empowerment, and job satisfaction among nursing staff in Malaysia.

Therefore, this research is focused on nursing staff, i.e., medical assistants and nurses, in the selected large private and public hospitals in Malaysia and explores the relationship among transformational leadership, empowerment, and job satisfaction. The purposes of this study were to (1) examine the influence of transformational leadership on job satisfaction, (2) investigate the influence of transformational leadership on empowerment, (3) investigate the influence of empowerment on job satisfaction, and (4) explore the mediating effect of empowerment between transformational leadership and job satisfaction. The mediating analysis is useful in predicting how the causal effect of transformational leadership on job satisfaction is intervened by employee empowerment and thus has tangible policy implications [19].

The study is based on a standardized survey to identify perceived transformational leadership traits, empowerment, and job satisfaction among nursing staff in the two studied hospitals. Purposive sampling was used and 200 valid samples were obtained. We sought to offer new evidence to highlight the importance of transformational leadership and empowerment in human resource development in the nursing profession in Malaysia. Specifically, transformational leadership has the potential to become a key strategic consideration for the ETP to build and retain qualified and skilled nurses in the healthcare industry.

### Transformational leadership

Transformational leadership was first conceptualized by Burns [20] and was then further developed by Bass [21]. In the current literature, the term tends to refer to Bass’ transformational leadership theory. According to Bass [22], there are four characteristics of transformational leaders. The first characteristic, individualized consideration suggests that transformational leaders support the development of subordinates’ skills and assist subordinates in achieving desired outcomes. Such leaders not only offer coaching and advices but also give employees attention and treat them as individuals. Second, transformational leadership includes intellectual stimulation, whereby leaders promote a culture in which employees will develop intelligence and rational thinking. Intellectual stimulation, in turn, fosters independent problem solving by employees. Inspiration is the third element of transformational leadership. In this regard, leaders communicate high expectations and encourage employees to focus their efforts on achieving established goals. To do this, transformational leaders tend to use effective communication techniques, such as symbols and simple language, to ensure that employees understand the main purposes of the assigned tasks. Finally, transformational leaders are regarded as charismatic leaders who offer a vision and a mission to employees. Such leaders will try to instill pride and gain respect and trust from employees so that the organization can achieve the required outcomes.

Many leadership scholars have agreed that transformational leadership plays a significant role in enhancing employee performance, trust, and commitment in organizations with a hierarchical authority structure [23–25]. The reason for this significance is that transformational leadership can be understood as a process of creating a vision and delivering a sense of belonging to employees [26]. Transformational leadership causes employees to perceive that the organization supports them and leads to attachments among the organization’s members. Such leadership establishes a strong relationship between employees and the organization, which supports organizational purposes. In short, transformational leadership builds a mission-oriented culture within an organization through a social influence process among organizational members [27, 28].

### Job satisfaction

Job satisfaction can be manifested as employee commitment that results from an increased sense of meaningfulness at work and improved accomplishments [29, 30]. Job satisfaction reflects employee perceptions of job performance. Employees with high levels of job satisfaction will feel that they are contributing positive value and outcomes to the organization. They also feel that they have a clear understanding of their job contribution. In addition, satisfied employees tend to perceive that they are treated fairly both inside and outside of an organization. In short, employees’ positive perceptions of their jobs and their organization can be revealed through job satisfaction.

Job satisfaction is a valuable indicator that management can use to assess overall employee development within an organization. Most satisfied employees tend to have very high self-confidence, which boosts their performance [31, 32]. Job satisfaction is linked to the employees’ willingness to develop work skills and personalities because they can sense whether the organization is concerned about their well-being. Job satisfaction cannot be ignored if improving job performance is a priority for management. Individual personalities are often unique, and thus, employees’ expectations regarding their jobs differ across individuals. Individual consideration is therefore important for motivating employees to achieve better job performance. Likewise, Luthans [32] suggests that job satisfaction is closely related to employees’ positive emotional state. The perceived state is often a result of whether employees sense that they will gain in terms of personal development through a job experience. A study by Stup [33] also suggests that employees who perceive that they are treated fairly by leaders tend to value the organizational structure. As a result, employees will have stronger trust in and attachment to the organization, as well as show higher job satisfaction.

### Empowerment

Employee empowerment has been a topic of discussion for many years. Several scholars have cautioned that empowerment that may alter the power distribution structure in an organization is a double-edged sword [34, 35]. To a certain extent, employee empowerment may be counterproductive to an organization. The rationale for this concern is that the implementation of empowerment practices signifies that a certain amount of authority and autonomy is given to employees. Some employees may become overconfident, and this false confidence will lead to management losing control over certain employees. These employees may abuse their power owing to misjudgments in their work. A good example of the potential downside of empowerment practices is when employees do not abide by corporate information management procedures when they are given access to confidential information [36, 37], i.e., reduced monitoring and supervision increase the possibility that information will be leaked to outsiders.

As stated above, management may lose control of employees if empowerment is not properly executed. However, scholars generally acknowledge that employee empowerment enhances job performance (e.g., [38, 39]). Empowerment is a strategic management option that can encourage employees to work beyond the norm and accomplish jobs in a flexible manner [38]. Such job flexibility is a precondition to instilling the decision-making ability of employees to respond swiftly to satisfy customer demands. More importantly, empowerment can stimulate employees’ attachment to their jobs because employees perceive the grant of decision-making authority as in indication that the organization appreciates their job contributions [39]. In short, job attachment is formed when employees associate positive emotions and acceptance with the organization.

### Relationship between transformational leadership and job satisfaction

Transformational leaders are generally described as leaders who transform the values, desires, aspirations, and priorities of their employees and motivate employees to outperform expectations [40]. The link between transformational leadership and job satisfaction is well established in the current literature [41]. The characteristics of transformational leadership conceptualized by Bass [22] provide theoretical foundation for explaining employees’ job satisfaction.

Bogler [42] explains that transformational behaviors can affect job satisfaction through employees’ perceptions of transformational leaders. Such leaders increase employees’ expectations and recognition of their work and enhance employees’ job satisfaction through transformational leadership behaviors such as individual attention, intellectual stimulation, and motivation. Additionally, the participative decision-making style practiced by transformational leaders gives employees a sense of involvement. Thus, employees are more committed to their jobs and have higher levels of job satisfaction. In the same vein, the study by Nemanich and Keller [43] suggests that job satisfaction occurs when employees are valued through transformational leadership behaviors, namely individual consideration and inspiration. This relationship can be understood as a reciprocal exchange because employees gain job satisfaction and become committed to producing better job outcomes when they are valued by organizations.

Individual and team perceptions of transformational leadership are also positively related to job satisfaction [44]. Transformational leaders not only consider their followers individually but also recognize the importance of the team. This dual consideration is evidenced through transformational behaviors, such as motivation and inspiration, that are tailored to both the individual and the entire team. For example, transformational leaders will communicate a vision and demonstrate considerate behavior to encourage all team members to work together to achieve organizational goals. Furthermore, interpersonal conflicts can be reduced when individuals work together as a team; the job satisfaction of both individuals and the entire team will be strengthened.

The relevance of transformational leadership to employees’ job satisfaction is not restricted to a particular organizational setting. Prior studies have consistently found that transformational behaviors occur and enhance followers’ job satisfaction in various organizational settings, including educational, industrial, military, and volunteer settings [44–48]. For instance, Yang et al. [47] found that followers’ positive perceptions of transformational behaviors by leaders (or supervisors) lead to stronger identification with the organization, increased internalization of organizational goals, and improved job satisfaction.

As stated previously, the role of transformational leadership in enhancing employees’ organizational commitment and job satisfaction cannot be denied. The effect of transformational leadership is important for individuals who work in rapidly changing environments (for example, R&D personnel in technology-based organizations) to strengthen their organizational commitment and job satisfaction [45]. Indeed, transformational leadership is important to any organization that experiences environmental changes, including public sector organizations, which are commonly perceived as undergoing minimal organizational change. In a similar vein, a study by Wright and Pandey [25] suggests that transformational leadership behaviors are not limited by procedural constraints and rules in organizations with hierarchical authority structures. Such organizations can opt to change leadership styles even if their hierarchical decision-making structures may constrain transformational leadership behaviors.

In particular, scholars have acknowledged the importance of transformational leadership in enhancing the job satisfaction of staff in healthcare industries [49–53]. Employees in the healthcare sector often work in high-pressure environments. Supervisors’ transformational behaviors can establish a sense of self-control and competence among employees and thereby enhance job satisfaction [49]. Andrews and Dziegielewski [52] explain that nursing staff generally prefer supervisors with transformational behaviors that address employees’ individual needs. Thus, transformational leadership can reduce nursing staff turnover owing to low job satisfaction. This reasoning leads to the following hypothesis:

H1: Transformational leadership has a positive impact on job satisfaction among medical assistants and nurses in Malaysia.

### Relationship between transformational leadership and empowerment

Empowerment is one of the mechanisms used to promote employee development in an organization’s long-term plan. Prior studies suggest that Bass’ [22] four characteristics of transformational leaders serve as antecedents to employee empowerment in organizations (e.g., [54–59]). Transformational leaders are persuasive and able to instill positive organizational perceptions among employees [60].

The charisma characteristic is considered to be a determinant of empowerment. Previous works suggest that charismatic leaders can intensify employee empowerment initiatives by offering vision and a sense of job ownership, as well as creating synergy and a climate of trust that fosters team spirit [54, 60–62]. Additionally, charismatic leaders encourage employees to participate in the decision-making process, which will encourage employees to continuously develop skills and knowledge. Employees’ sense of responsibility is thus intensified. Employees will gain self-confidence and job-specific technical skills, and a sense of psychological identification is created. In sum, charismatic leaders use empowerment to foster employees’ sense of psychological identity with the organization.

Transformational leaders also use intellectual stimulation to empower employees. Sharing certain decision-making powers with employees is a precondition to promoting intellectual stimulation [55, 56]. Employees who are granted decision-making power tend to repay the trust given to them by leaders by achieving organizational goals [63]. The delegation of power gives employees the sense that they are valued by the organization. Employees who value their leaders’ command will establish strong leader-employee relationships. A similar argument can be detected not only in the theoretical literature but also in empirical experiments. Prior studies have shown that transformational leadership has a positive impact on employee empowerment [24, 54, 55, 61].

Individual consideration is equally important to encourage employees to accept an empowerment initiative [57]. Employees tend to be motivated when transformational leaders give them individual attention and build a coaching system to individually develop employees’ expertise. Such individual consideration can stimulate a productive working environment in an organization. Moreover, employees highly value their jobs when they are treated fairly and valued by leaders [60].

Notably, a recent study shows that transformational leaders tend to stimulate the acceptance of empowerment by employees [64]. All of the elements of transformational leadership conceptualized by Bass [22] are highly and positively correlated with empowerment success. In other words, these elements are valued as universal components of transformational behavior to empower employees in the workplace. The following hypothesis is thus proposed:

H2: Transformational leadership has a positive impact on employee empowerment among medical assistants and nurses in Malaysia.

### Relationship between empowerment and job satisfaction

Employee empowerment is important for curbing workplace stress among employees [38]. The role of empowerment in enhancing job satisfaction is manifested by employees’ perceived job attachments and thus their reduced stress [39]. For example, when employees are given decision-making power, positive emotions and employee acceptance of the organization intensify. Empowered employees develop a climate of trust with their leaders and become more creative and innovative. Empowerment may foster critical thinking, which leads to employees working at a higher level. Such empowerment positively shapes employees’ perceptions of their jobs, reduces stress, and eventually leads to higher job satisfaction.

Those who work in healthcare industries largely view their working environment as stressful [49]. Work-related stress can lead to burnout and decreased job satisfaction. Empowerment can be viewed as an organizational initiative to give autonomy to employees for the purpose of diminishing feelings of powerlessness and removing formal barriers in the organizational environment [35]. It is believed that nursing staff must often respond to patient needs through rapid decision-making and thus the elimination of formal barriers is important. Organizations that embrace empowerment by sharing the decision-making process with their nursing staff will alleviate workplace-related stress [35, 65, 66]. This proposition leads to the next hypothesis:

H3: Employee empowerment has a positive impact on job satisfaction among medical assistants and nurses in Malaysia.

**Fig. 1:**
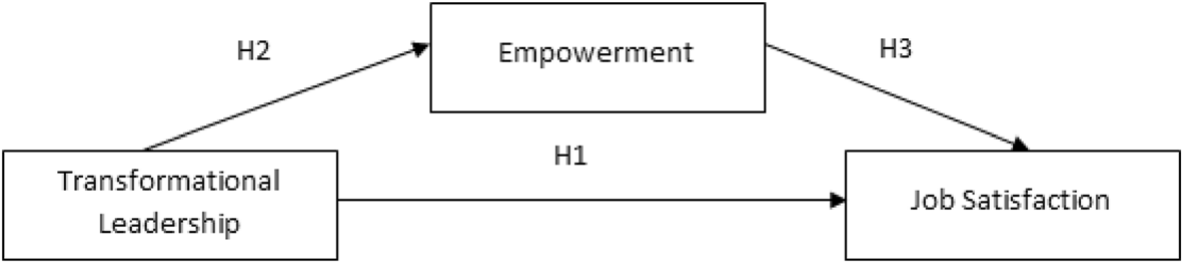
Research framework

The conceptual framework has been constructed based on the hypotheses presented above regarding transformational leadership, empowerment, and job satisfaction (see Fig. 1). The framework indicates that empowerment is a mediator that exerts an intervening effect on the relationship between transformational leadership and job satisfaction.

## Methods

### Study design and sample selection

The population of interest was selected by sampling during several stages. Table 1 presents statistics regarding hospitals and nurses in Malaysia. In 2014, there were 326 hospitals, 92 681 nurses, and 12 773 medical assistants in 13 Malaysian states. We initially selected the state of Johor because it is a prominent health tourism hub in Malaysia [67, 68]. Johor is comprised of 10 districts, and we purposely selected Johor Bahru as the studied region. Johor Bahru is the second most populous state located in the southern part of Malaysia. Johor Bahru is known as the South Johor Economic Region and is a major corridor for economic development under the ETP [69]. There are three public and five private hospitals in Johor Bahru. A large private hospital and a large public hospital were chosen for this study. Both private and public hospitals are perceived as having stressful working conditions, but the nursing staff workload is heavier in public hospitals than in private hospitals, which is the result of the universal subsidization of public healthcare services in Malaysia [70].

**Table 1:** Statistics regarding hospitals, nurses, and medical assistants in Malaysia

Year	Public hospital	Private hospital	Public hospital		Private hospital		Total number		Profession: population	
			Nurse	Medical assistant	Nurse	Medical assistant	Nurse	Medical assistant	Nurse	Medical assistant
2008	136	209	45 060	8 648	14 315	766	59 375	9 414	1:477	1:3 007
2009	137	217	47 992	9 556	21 118	794	69 110	10 350	1:410	1:2 738
2011	138	220	50 063	10 289	24 725	873	74 788	11 162	1:387	1:2 595
2012	140	209	56 089	10 902	28 879	944	84 968	11 846	1:345	1:2 477
2013	141	214	56 503	10 641	32 664	1 867	89 167	12 517	1:333	1:2 374
2014	142	184	59 364	11 305	33 317	1 468	92 681	12 773	1:325	1:2 356

Source: Ministry of Health Malaysia. The Ministry of Health Malaysia does not release the report on year 2010

The questionnaires were written in English. All questionnaires were administered face-to-face and were collected on site by one of the researchers within a 1-month period. During the data collection, the respondents were allowed to end the interview if they did not wish to answer certain questions. The researcher approached 350 nurses and medical assistants employed by the private and public hospitals selected for this study. However, 150 nurses and medical assistants either refused to answer the questionnaires or terminated their participation during the interview. For the latter case, we considered the incomplete surveys as invalid responses and that they were unwilling to participate in our study. In total, 101 and 99 valid responses (completed questionnaires) were obtained from the nursing staff of the public and private hospitals, respectively. The effective response rate was 57.14%.

This study was approved by the Commercialization & Technology Management Group (CTMG), a multidisciplinary research unit of the SmartDigital Community Research Alliance of Universiti Teknologi Malaysia, Malaysia. One of the researchers contacted the hospitals for permission to distribute the questionnaires. The names of the hospitals were not disclosed in accordance with the hospitals’ requests, and we believe that respondents prefer such information to be kept confidential. Furthermore, the questionnaires did not request the working position to be reported because it may reveal identifying information of medical assistants, as their proportion is significantly smaller than that of nurses. The respondents were aware of and willing to participate in this study. We assured the respondents of confidentiality before they completed the survey.

### Survey instruments

A set of questionnaires was developed to measure the constructs in this conceptual model. The three constructs are transformational leadership (eight items), empowerment (five items), and job satisfaction (four items). Existing measurements for multi-item constructs that have been verified in the literature were used when possible. A 5-point Likert scale was used, ranging from 1 (strongly disagree) to 5 (strongly agree), to measure negative and positive assessments of the trait. Three survey instruments—transformational leadership, empowerment, and job satisfaction constructs—were used. The transformational leadership instrument was adapted from the Multifactor Leadership Questionnaire (MLQ) form developed by Bass and Avolio [71]. The transformational leadership instrument measures charisma, inspirational motivation, intellectual stimulation, and charisma exhibited by supervisors. The empowerment instrument was adapted from Matthews et al. [72], and the job satisfaction instrument was adapted from Warr et al. [73]. The empowerment instrument measures the perceived control of workplace decisions, the dynamic structural framework, and the fluidity in information sharing within the organization. The job satisfaction instrument measures the perceived compensation and the recognition dimensions among nursing staff. These instruments measure self-awareness of the nursing staff with respect to the transformational leadership, empowerment, and job satisfaction; thus, some discrepancies may exist if compared to external assessment.

All survey instruments were first reviewed by two experts to obtain feedback on their validity and clarity in the Malaysian context. The first expert, an academician whose research expertise is in human resource management, was asked to evaluate the content validity of the questionnaire. The second expert was a professional, a senior nurse, who evaluated the face validity of the questionnaire. The survey instruments were slightly modified based on feedback from the experts. The final questionnaire has four sections: the first section examines the demographic profiles of respondents; the second section investigates transformational leadership; the third section examines employee empowerment; and the final section investigates job satisfaction.

### Statistical analysis

Data analysis was conducted using PASW Statistics 18.0 and SmartPLS 3.0. A descriptive analysis using PASW Statistics 18.0 was conducted to summarize the demographic backgrounds of the respondents. SmartPLS 3.0 was used to perform partial least squares-structural equation modeling (PLS-SEM) to validate the measurements and test the proposed hypotheses.

PLS-SEM is a second-generation regression technique for complex causal modeling, which is also known as variance-based structural equation modeling [74–77]. PLS-SEM is a causal modeling technique that is designed to maximize the explained variance of dependent variable(s). PLS-SEM is a preferred method when the research objective is prediction oriented. PLS-SEM also provides robust estimations of cause-and-effect-relationship models and/or when the collected data cannot meet certain assumptions (i.e., a small sample size and non-normal data). Compared to traditional regression techniques, PLS-SEM offers several significant advantages that suit our study [75, 78]. First, PLS-SEM is a causal modeling technique that simultaneously estimates the dual roles of the mediator: one as a causal variable in the outcome and the other as an intervening variable in the mediation model. Second, PLS-SEM is appropriate for this exploratory study, which entails developing new ideas to verify the mediating role of empowerment between transformational leadership and job satisfaction. Third, PLS-SEM allows the testing of higher-order models, which contain two layers of constructs. In this study, job satisfaction is conceptualized as a two-dimensional construct (or higher-order model) that can be explained by *recognition* and *pay*. Modeling job satisfaction as a higher-order model reduces the number of relationships between transformational leadership and job satisfaction and between empowerment and job satisfaction. Otherwise, one would be required to estimate the relationship between transformational leadership and empowerment for each dimension of job satisfaction. The higher-order model approach allows the path model to be more parsimonious and easier to comprehend [75]. The reflective-reflective type of higher-order model was used to reflect the two dimensions of job satisfaction in this study. Following the suggestion of Hair et al. [75], a repeated indicator approach was used for the higher-order model (i.e., for job satisfaction).

Using the latest guidelines for PLS-SEM (e.g., [74, 75, 79]), we followed a two-step approach for assessing the measurement and structural models. In the first step, the assessment began with the measurement model. The assessment is to ensure sufficient construct reliability (i.e., indicator reliability and internal consistency) and validity (i.e., convergent validity and discriminant validity) are achieved. Additionally, Harman’s single-factor test was used to investigate common method bias. The reason for conducting this test is that self-report surveys were used to collect data regarding job satisfaction and thus the data may be susceptible to common method variance. The perceptual measures of explanatory and dependent variables were derived from the same respondents at the same time and respondents may have a propensity to offer consistent or systematic answers to survey questions that are otherwise not related [80].

The second step is to assess the structural models. In this regard, a PLS algorithm was selected based on considerations regarding our research design. Path weighting scheme was selected as the PLS algorithm because it can be applied to virtually all kinds of path model specifications and estimations, including a path model with a higher-order model [75, 81]. A bootstrapping with 5000 samples was used to estimate the path coefficients’ significance in the path analysis [75]. A path analysis was performed for the structural model following the specific suggestion by Hair et al. [75] to perform the mediational analysis with PLS-SEM. Assessment was performed to ensure predictive relevance and the absence of multicollinearity in the structural models.

## Results

### Descriptive statistics

Of the responses received, only 200 were found to be valid for analysis. Table 2 presents the demographics of the respondents in the two studied hospitals. Females represented 92.0% of the respondents, which is not surprising because nursing is a female-dominated profession in Malaysia [9]. The age group that was most represented in the survey was 26 to 30 years of age (59%). The largest educational background group was undergraduate diploma (95.5%). Approximately 60.5% of respondents indicated that they have 2–5 years of working experience. The findings in this study can be generalized to the two studied hospitals but are not representative of total populations of nurses and medical assistants in Malaysia. Thus, results should be interpreted with caution.

**Table 2:** Descriptive analysis for the demographic background

Demographic	Items	Frequency	Percentage (%)
Characteristics			
Gender	Male	16	8.0
	Female	184	92.0
Age	20–24	28	14.0
	25–30	118	59.0
	30–35	48	24.0
	36 and above	6	3.0
Educational background	Diploma	190	95.5
	Bachelor degree	10	4.5
Working experience	1–2 years	34	17.0
	2–5 years	121	60.5
	5 years and above	45	22.5
Types of hospital	Private	99	49.5
	Public	101	50.5

### Measurement model

We assessed the construct reliability (i.e., indicator reliability and internal consistency) and validity (i.e., convergent validity and discriminant validity) for the measurement model (see Table 3). According to Hair et al. [74], the indicator loadings should be greater than 0.70, whereas loadings between 0.40 and 0.70 should be removed only if their deletion can increase the composite reliability to its minimum threshold value. Four indicator loadings ranged from 0.64 to 0.68, whereas all other indicator loadings were above 0.70. An analysis of the indicators with loadings less than 0.7 by deletion was conducted. Because the deletion of these indicators would not increase the respective composite reliability, the indicators were retained for this study. In short, the indicator loadings had satisfactory indicator reliability levels.

**Table 3:** Measurement models

Construct	Item	Loadings	Composite reliability	AVE
Transformational leadership (TL)	TL_1	0.68	0.892	0.508
	TL_2	0.65		
	TL_3	0.77		
	TL_4	0.74		
	TL_5	0.70		
	TL_6	0.70		
	TL_7	0.74		
	TL_8	0.72		
Empowerment (Emp)	Emp_1	0.64	0.876	0.542
	Emp_2	0.67		
	Emp_3	0.76		
	Emp_4	0.77		
	Emp_5	0.78		
Recognition	JS3	0.92	0.907	0.904
	JS4	0.90		
Pay	JS1	0.95	0.949	0.83
	JS2	0.95		
Job satisfaction (JS)^a^	JS_Reg	0.83	0.804	0.508
	JS_Pay	0.71		

^a^Higher-order construct

The assessment of the composite reliability showed that all constructs had a value greater than 0.7, which indicates sufficient internal consistency reliability [74]. An examination of the convergent validity and the discriminant validity was conducted to assess the validity of the constructs. First, the average variance extracted (AVE) of all constructs was greater than the minimum threshold value of 0.50, which verifies the convergent validity. Then, the study used the Fornell-Lacker criterion, which is a more conservative approach than cross-loadings, to assess discriminant validity [75]. The findings show that the discriminant validity is verified because the square root of the AVE of each construct is higher than its correlation with all other constructs (see Table 4).

**Table 4:** Discriminant validity of constructs

	Empowerment	Job satisfaction	Transformational leadership
Empowerment	**0.736**		
Job satisfaction	0.419	**0.713**	
Transformational leadership	0.649	0.406	**0.713**

The bold and diagonal values represent the square root of AVE whereas the off diagonals represent the correlations of constructs

It is important to note that the same evaluation criteria were utilized to evaluate the measurement model of the higher-order model (i.e., job satisfaction). As discussed above, the indicator loadings, composite reliability, and AVE for the higher-order construct of job satisfaction have satisfactory reliability and validity. In short, the measurement model assessment confirmed that all constructs, including the higher-order model, are reliable and valid.

Finally, Harman’s single-factor test was used to investigate common method bias. Four scaled constructs were loaded into a factor analysis. The results indicated that there are four factors present and that they account for 62.3% of the total explained variance. The highest load factor accounts for 37.4% of total variance explained. These results show that common method variance is not significant because no single factor emerges and no single factor accounts for the majority of the variances between measures [80, 82].

### Structural model

A step-by-step analysis was conducted to offer a thorough analysis. In the first step, the focus was on the relationship between transformational leadership and job satisfaction. Subsequently, the mediator (i.e., empowerment) was introduced, and the full structural model was assessed.

**Fig. 2:**
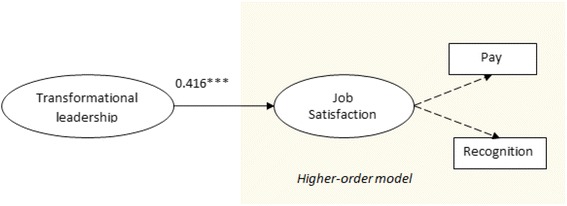
Structural model without the mediator

Figure 2 and Table 5 show the results of step 1 in the mediational analysis. First, an assessment of collinearity was performed to examine whether the predictor constructs were closely correlated with endogenous constructs. The (unreported) variance inflation factor (VIF) of the predictor constructs was below 3.0, indicating the absence of collinearity. Additionally, the *Q* ^2^ value generated by a blindfolding procedure was larger than zero, indicating the predictive relevance of the structural model [74]. The path analysis indicates that transformational leadership is positively related to job satisfaction (*p* < 0.01).

**Table 5:** Structural model assessment of model 1 (PLS path model without mediator)

Endogenous constructs	*R* ^2^	*Q* ^2^			
Job satisfaction	0.173	0.075			
Relation	Path coefficient	*t* value (bootstrap)	*p* value	Bias corrected 95% confidence intervals	
Transformation leadership → job satisfaction	0.416	7.012	0.000	0.358	0.597

**Fig. 3:**
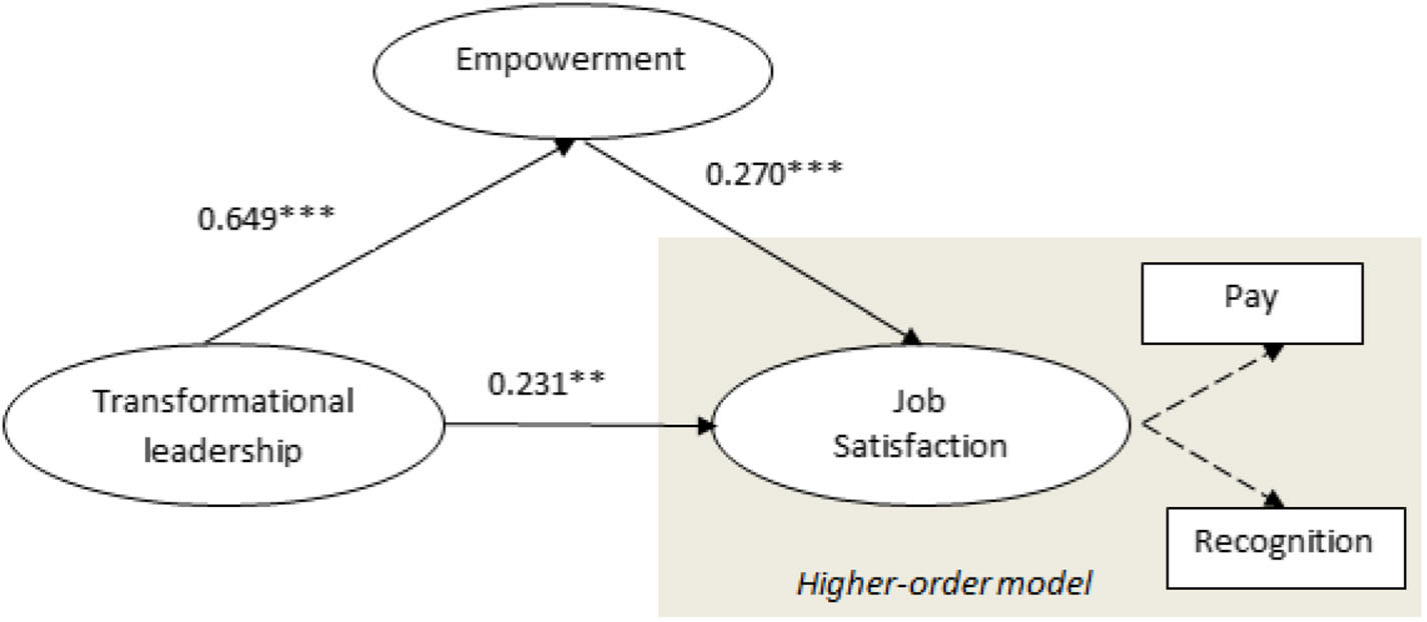
Full structural model with the mediator

Next, the full structural model was assessed by including the empowerment construct (see Fig. 3 and Table 6). The *Q* ^2^ of the full structural model was above zero, and the (unreported) VIF was less than 3 for all predictor constructs. The results show that the positive effect of transformational leadership on job satisfaction remains significant (*p* < 0.05). Similarly, transformational leadership exhibits a positive effect on empowerment (*p* < 0.01), and empowerment, in turn, positively affects job satisfaction (*p* < 0.01).

**Table 6:** Structural model assessment of model 2 (PLS path model with mediator)

Endogenous constructs	*R* ^2^	*Q* ^2^			
Empowerment	0.522	0.225			
Job satisfaction	0.207	0.086			
Relation	Path coefficient	*t* value (bootstrap)	*p* value	Bias corrected 95% confidence intervals	
Empowerment → job satisfaction	0.270	3.337	0.001	0.115	0.431
Transformation leadership → empowerment	0.649	16.401	0.000	0.589	0.741
Transformation leadership → job satisfaction	0.231	2.485	0.013	0.075	0.438

It is important to note that the relationship between transformational leadership and job satisfaction is significant in steps 1 and 2 but with a difference of 0.185. These results indicate that the mediator (i.e., empowerment) may absorb some effect of the relationship between transformational leadership and job satisfaction. Table 7 presents the mediating effect results, which show that the indirect effect is significant (*p* = 0.001, *t* value = 3.28). These results show that the variance accounted for (VAF) is 43.1%, which indicates that a partial mediating effect exists [75].

**Table 7:** Analysis of mediating effect

Relation	Direct effect	Indirect effect	Total effect	VAF
Transformation leadership → job satisfaction	0.231	0.175	0.406	43.1%

## Discussion

The Malaysian healthcare industry has experienced significant growth in recent years under the ETP, but human capital development in the healthcare sector remains a significant challenge. The purpose of this study is to investigate the causal relationships among perceived transformational leadership, empowerment, and job satisfaction amid nurses and medical assistants in the selected large private and public hospitals in Malaysia. This study also performed causal mediation analysis to identify the mediating effect of empowerment on the relationship between transformational leadership and job satisfaction.

First, we tested the hypothesis of whether transformational leadership has a positive impact on job satisfaction among medical assistants and nurses in two studied hospitals in Malaysia. The results show that transformational leadership positively affects job satisfaction and hypothesis H1 is accepted. Our results suggest that the perceived transformational leadership behaviors enhance job satisfaction among medical assistants and nurses. As such, our findings are consistent with correlation analyses from previous studies that investigated the relationship between transformational leadership and job satisfaction among nurses in Malaysia [15, 16]. However, our findings offer the first empirical evidence to validate the causal impact of transformational leadership on job satisfaction among medical assistants and nurses in two studied hospitals in Malaysia.

The second hypothesis (H2) proposed that transformational leadership has a positive impact on employee empowerment among medical assistants and nurses in the two studied hospitals. The results indicate that the hypothesis is supported by the data. Our study presents empirical evidence that supports the shared view in workplace literature that transformational leadership can enhance employee empowerment within a hierarchical structure [15, 24, 45, 46, 54, 55, 83, 84]. In this study, transformational leaders are those who exhibit charisma and have abilities to inspire and intellectually stimulate subordinates, not only individually but also as a team. Based on our findings, it appears that transformational leadership led to medical assistants and nurses to establishing a stronger sense of self-determination and competency, which could, in turn, significantly impact their work and job satisfaction.

The third hypothesis (H3) proposed that employee empowerment has a positive impact on job satisfaction among medical assistants and nurses in the studied hospitals. The results show that empowerment has a positive and significant influence on job satisfaction. The findings are consistent with several studies that found positive influence of empowerment on job satisfaction among nurses in Malaysia and other countries [11, 17, 18]. Such findings could be attributed to the role of employee empowerment in restructuring work environment that reducing the powerlessness senses among nursing staff in this study [9, 11, 12, 17, 18]. We believe that the empowerment effect may be more evident among Malaysian nursing staff where nursing is regarded as a low status and oppressed profession [9]. Thus, empowerment through mechanisms such as shared decision-making processes and improved autonomy can reduce the sense of powerlessness among nursing staff in an oppressive work environment. When nursing staff perceive that they are empowered, powerlessness and job burnout are mitigated, which results in higher job satisfaction.

The final step in the analysis was the examination of the causal mediation analysis to identify whether empowerment mediates the relationship between transformational leadership and job satisfaction among medical assistants and nurses in the studied hospitals. The results suggest that the indirect effect is significant, and partial mediating occurs, i.e., the employee empowerment explained the relationship between transformational leadership and job satisfaction.

### Limitations and delimitations

The findings of this study should be interpreted with caution due to empirical design considerations. First, this study only investigates medical assistants and nurses from two hospitals in Malaysia, and thus, results are not representative of the entire Malaysian nursing workforce. Second, our sample size was not sufficient to disaggregate the analysis in order to identify the masking discrepancies between male and female nursing staff. Finally, this study uses a cross-sectional design, which makes it difficult to determine temporal relationships.

It is important to note that the response rate of the current study was 57.14%. The rate was caused by the distinctive sampling procedure in this study, i.e., potential respondents who were approached in this study were notified of their rights to refuse to participate or terminate the interview if they did not wish to answer certain questions. The majority of nursing staff who refused to participate in our study stated that they were busy whereas others did not give any reason. During the interview, the researchers would ask whether the nursing staff would like to terminate their participation if they seemed reluctant or uncomfortable to answer questions. Such approach was to ensure that they were treated with dignity and respect. Based on the observation during the data collection, there were no specific characteristics of nursing staff who refused to participate or terminated their participation during the interview. Similarly, there were no specific characteristics of those respondents who completed the interview. Nevertheless, it is impossible to completely rule out sampling bias that may be stemmed from distinctive sampling procedure in this study.

### Theoretical and practical implications

This study has several theoretical implications. Theoretically, this study is the first in the nursing management literature to develop and verify a theoretical framework for the relationships between the perceptions of transformational leadership, empowerment, and job satisfaction. This study offers a more thorough understanding of what drives the level of job satisfaction among nursing staff. Prior studies have clearly indicated the direct positive effect of transformational leadership on employees’ job satisfaction in the healthcare industry [49–52] but lack a deeper understanding of the role of empowerment that underlies this relationship. The causal mediation analysis in this study confirms that empowerment mediates the relationship between transformational leadership and job satisfaction among medical assistants and nurses in two selected hospitals. In other words, employee empowerment cannot be detached when investigating transformational leadership and job satisfaction phenomena in nursing management. Second, this study developed and carried out field testing of an instrument for measuring three constructs, i.e., transformational leadership, empowerment, and job satisfaction, in nursing management literature. Third, this study achieved aforementioned theoretical contributions in nursing management literature in an understudied Malaysian context [5, 12]. Finally, this study offers a baseline model for the role of transformational leadership in empowerment and job satisfaction in an institutional healthcare context. Because the oppression of nurses is a global phenomenon [9, 85], we hope that our research may trigger an examination of the use of transformational leadership and empowerment as possible practices in human resources to enhance job satisfaction and increase the retention of nursing staff, especially in countries with similar institutional contexts, such as developing and Islamic countries.

This study also offers important insights for healthcare managers to implement policies to enhance job satisfaction among medical assistants and nurses. The implications of our study are aligned with the call from the World Health Organization to enable evidence-based policy-making for human resources for health in Malaysia [3]. First, job satisfaction can be altered if hospital administrators promote transformational leadership practices. However, raising transformational leadership practices are insufficient because employee empowerment will mediate the positive effect of transformational leadership on job satisfaction. From the perspective of the nursing staff, empowerment can be an indicator of organizational intent to give them autonomy by removing formal organizational barriers [35]. The ability of nursing staff to offer more rapid responses through decision-making will alleviate the work-related stress that causes job burnout and will thus contribute to higher job satisfaction. Furthermore, from a managerial perspective, employee empowerment can be an effective mechanism to promote employee development that is aligned with organizational goals. In short, transformational leadership and empowerment are the two essential ingredients that have tangible policy implications to address low job satisfaction among medical assistants and nurses especially in the two studied hospitals.

Finally, our empirical evidence can be a useful input for Malaysian policymakers to revamp human resources’ policies for healthcare under the ETP. The job satisfaction of healthcare workers ensures that the nursing profession will achieve its full potential, thereby safeguarding an effective health care delivery system [66, 86]. In particular, our study highlights the important role of transformational leadership and empowerment in enhancing job satisfaction among medical assistants and nurses, which should be a focus of the Malaysian healthcare system under the ETP.

## Conclusions

This study was designed to address a knowledge gap in nursing management research regarding the causal relationships among perceived transformational leadership, empowerment, and job satisfaction amid medical assistants and nurses in the selected hospitals in Malaysia. Additionally, causal mediation analysis was performed to examine whether empowerment mediates the relationship between transformational leadership and job satisfaction. The findings of this study suggest that transformational leadership positively influences job satisfaction among medical assistants and nurses in the studied hospitals. This study also shows that employee empowerment is indispensable for enhancing job satisfaction. Thus, the empowerment factor not only positively affects job satisfaction but also mediates the relationship between transformational leadership and job satisfaction. Overall, the findings suggest that policy intervention must cover both transformational leadership and empowerment to enhance job satisfaction among medical assistants and nurses.
